# Emergence of an IncX3 plasmid co-harbouring the carbapenemase genes *bla*_NDM-5_ and *bla*_OXA-181_

**DOI:** 10.1093/jacamr/dlae073

**Published:** 2024-05-13

**Authors:** Hui Zuo, Yo Sugawara, Kohei Kondo, Shizuo Kayama, Sayoko Kawakami, Kohei Uechi, Ami Nakano, Koji Yahara, Motoyuki Sugai

**Affiliations:** Antimicrobial Resistance Research Center, National Institute of Infectious Diseases, Tokyo, Japan; Antimicrobial Resistance Research Center, National Institute of Infectious Diseases, Tokyo, Japan; Antimicrobial Resistance Research Center, National Institute of Infectious Diseases, Tokyo, Japan; Antimicrobial Resistance Research Center, National Institute of Infectious Diseases, Tokyo, Japan; Antimicrobial Resistance Research Center, National Institute of Infectious Diseases, Tokyo, Japan; Division of Clinical Laboratory and Blood Transfusion, University of the Ryukyus Hospital, Okinawa, Japan; Division of Clinical Laboratory and Blood Transfusion, University of the Ryukyus Hospital, Okinawa, Japan; Antimicrobial Resistance Research Center, National Institute of Infectious Diseases, Tokyo, Japan; Antimicrobial Resistance Research Center, National Institute of Infectious Diseases, Tokyo, Japan

## Abstract

**Background:**

The spread of transmissible plasmids with carbapenemase genes has contributed to a global increase in carbapenemase-producing Enterobacterales over the past two decades, with *bla*_NDM_ and *bla*_OXA_ among the most prevalent carbapenemase genes.

**Objectives:**

To characterize an *Escherichia coli* isolate co-carrying *bla*_NDM-5_ and *bla*_OXA-181_ (JBEHAAB-19-0176) that was isolated in the Japan Antimicrobial Resistant Bacterial Surveillance in 2019–20, and to evaluate the functional advantage of carrying both genes as opposed to only one.

**Methods:**

The whole-genome sequence of the isolate was determined using long- and short-read sequencing. Growth assay and co-culture experiments were performed for phenotypic characterization in the presence of different β-lactam antibiotics.

**Results:**

WGS analysis showed that *bla*_NDM-5_ and *bla*_OXA-181_ were carried by the same IncX3 plasmid, pJBEHAAB-19-0176_NDM-OXA. Genetic characterization of the plasmid suggested that the plasmid emerged through the formation of a co-integrate and resolution of two typical IncX3 plasmids harbouring *bla*_NDM-5_ and *bla*_OXA-181_, which involved two recombination events at the IS*3000* and IS*26* sequences. When cultured in the presence of piperacillin or cefpodoxime, the growth rate of the transformant co-harbouring *bla*_NDM-5_ and *bla*_OXA-181_ was significantly higher than the transformant with only *bla*_NDM-5_. Furthermore, in co-culture where the two *bla*_NDM-5_-harbouring transformants were allowed to compete directly, the strain additionally harbouring *bla*_OXA-181_ showed a marked growth advantage.

**Conclusions:**

The additional carriage of *bla*_OXA-181_ confers a selective advantage to bacteria in the presence of piperacillin and cefpodoxime. These findings may explain the current epidemiology of carbapenemase-producing Enterobacterales, in which bacteria carrying both *bla*_NDM-5_ and *bla*_OXA-48_-like genes have emerged independently worldwide.

## Introduction

There has been a global emergence of carbapenem-resistant Enterobacterales with escalating resistance to all available β-lactam antibiotics, which WHO has highlighted as critical priority pathogens.^1^ The major mechanism underlying carbapenem resistance is the degradation of carbapenems via bacterial enzymes called carbapenemases. One such enzyme is NDM, which belongs to the Ambler class B β-lactamases and is encoded by *bla*_NDM_, which confers resistance not only to carbapenems but also to almost all β-lactams.^2^ The *bla*_NDM_ gene is disseminated worldwide, predominantly in Asia,^2^ and is usually carried by transferable plasmids, such as IncX3-type plasmids, enabling it to spread broadly among species.^3–5^ The OXA-48 enzyme is also a carbapenemase of concern. It is difficult to detect because of the low-level *in vitro* resistance to carbapenem antibiotics it confers, posing a challenge to clinical laboratories.^6,7^ Genes encoding OXA-48-like enzymes, such as *bla*_OXA-48_, *bla*_OXA-181_ and *bla*_OXA-232_, are globally widespread.^6,7^ The *bla*_NDM_ and *bla*_OXA-48_-like genes were identified as the second and third most prevalent carbapenemase genes among carbapenemase-producing Enterobacterales (CPE), respectively, in a global surveillance programme conducted between 2008 and 2014.^8^ Specifically, *bla*_OXA-181_ and *bla*_NDM-5_ were the first and second most prevalent carbapenemase genes, respectively, among global carbapenemase-producing *Escherichia coli* strains collected in 2015–17.^9^ Although the sole presence of *bla*_NDM_ confers sufficiently high-level carbapenem resistance to bacteria, there have been many previous reports on isolates co-carrying *bla*_NDM_ and *bla*_OXA-48_-like genes.^10^ Most harbour these two genes on two separate plasmids, whereas only a few co-harbour them on the same plasmid, such as the *Acinetobacter* plasmid GR59.^11^ Here, we report an IncX3 plasmid co-harbouring *bla*_NDM-5_ and *bla*_OXA-181_ and explore the potential advantage of carrying both of these genes through comparative phenotypic analysis with typical endemic IncX3 plasmids harbouring only one of the two genes.

## Materials and methods

### Bacterial isolates and antimicrobial susceptibility testing

An *E. coli* isolate, JBEHAAB-19-0176, was isolated during the Japan Antimicrobial Resistant Bacterial Surveillance, focusing on Gram-negative bacteria (JARBS-GNR) that had been conducted during 2019–20, which also focused on nosocomial Enterobacterales isolates with reduced carbapenem susceptibility and/or resistance to third-generation cephalosporins.^12^ The current study was approved by the International Review Board of the National Institute of Infectious Diseases (approval number: 1553). The isolate was recovered from the faeces of an outpatient at the University of Ryukyus Hospital (Okinawa, Japan). Antimicrobial susceptibility testing was performed using the MicroScan WalkAway Plus system with Neg MIC EN 2J and 3.31E panels (Beckman Coulter, Brea, CA, USA). The MICs of meropenem, imipenem, ampicillin, piperacillin and cefpodoxime were further determined using a broth microdilution method, according to the CLSI guidelines.

### Bioinformatics and plasmid replicon analysis

Genomic and plasmid DNA were extracted using a Monarch HMW DNA Extraction Kit for Tissues (New England Biolabs, Ipswich, MA, USA) and a QIAGEN Plasmid Mini kit (QIAGEN, Hilden, Germany), respectively, and WGS data obtained using HiSeq X (Illumina, San Diego, CA, USA) and GridION systems (Oxford Nanopore Technologies, Oxford, UK), as previously described.^12^ Obtained raw reads were assembled, as previously described,^12^ and antimicrobial resistance gene detection and plasmid Inc typing performed using ResFinder v2.1^13^ and PlasmidFinder v1.3,^14^ respectively. Plasmid sequences were annotated using DFAST v1.2.0^15^ and compared using BLAST (http://blast.ncbi.nlm.nih.gov) and Easyfig v2.2.3.^16^ Transposon and insertion sequences were identified using ISfinder.^17^

### Transformation and conjugation

Plasmid DNA was introduced into *E. coli* HST08 cells (Takara Bio, Shiga, Japan) via electroporation using a Gene Pulser Xcell (Bio-Rad, Hercules, CA, USA). The size of the plasmids in the transformants and the presence of *bla*_NDM-5_ and *bla*_OXA-181_ on them were confirmed through S1 nuclease digestion of whole genomic DNA, followed by PFGE and Southern hybridization, as described previously.^5^ The conjugation assay was conducted as previously described, with slight modifications,^5^ using rifampicin-resistant *E. coli* ML4909^18^ as the recipient. Transformants carrying IncX3 plasmids were mixed with recipient cells in a 1:10 ratio and incubated on a nitrocellulose membrane filter at 37°C for 2 h. The bacterial mixture was then suspended in brain heart infusion (BHI) broth and plated onto BHI agar containing meropenem (0.125 mg/L) and rifampicin (100 mg/L). The presence of *bla*_NDM-5_ and/or *bla*_OXA-181_ in the colonies was confirmed using colony-direct PCR. The conjugation frequency was calculated by dividing the number of cfu of the transconjugants by the number of cfu of the donor and transconjugants.

### Growth curve and pairwise competition assay

Growth rates were determined according to a previously published study,^19^ with a few modifications. Briefly, bacterial suspensions of pJBEHAAB-19-0176_NDM-OXA and pJBBDAGF-19-0019_NDM-5 transformants were adjusted to OD_600_ = 0.5 and then diluted 100-fold in LB broth containing piperacillin (64 mg/L), cefpodoxime (64 mg/L), meropenem (128 mg/L), ampicillin (2048 mg/L), cefazolin (512 mg/L), cefoxitin (512 mg/L), flomoxef (256 mg/L), ceftazidime (512 mg/L), cefotaxime (512 mg/L), cefepime (128 mg/L) or imipenem (128 mg/L). Drug concentrations were set to maximize any differences in growth rates. The OD_600_ was measured for 36 h at 37°C with vigorous shaking (800 rpm) using a LogPhase 600 Microbiology Reader (Agilent Technologies, Santa Clara, CA, USA).

Competition assays were conducted as previously described,^19^ with slight modifications. Bacterial suspensions of the above two transformants were adjusted to OD_600_ = 0.5 and mixed at a 1:1 ratio. The mixture was then diluted 100-fold in LB broth with or without piperacillin (128 mg/L), cefpodoxime (64 mg/L) or meropenem (16 mg/L) and incubated at 37°C with shaking. The number of cells in each culture was evaluated at 24 h timepoints by spreading serially diluted cells and then culturing them on two types of BHI agar plates. One plate contained 0.25 mg/L meropenem to count the number of the two transformants, and the other plate contained 0.25 mg/L meropenem and 0.125 mg/L levofloxacin to count the number of pJBEHAAB-19-0176_NDM-OXA transformants carrying the quinolone resistance gene *qnrS1*. The competition indices were calculated by dividing the number of pJBEHAAB-19-0176_NDM-OXA transformants by the total number of transformants.

### Plasmid construction and induction of bla_OXA-181_ expression

The *bla*_OXA-181_ gene was cloned into the arabinose-inducible expression vector, pBAD18-cm,^20^ as described previously.^21^ The pBAD18-cm and the one harbouring *bla*_OXA-181_ were introduced into *E. coli* HST08 carrying the pJBBDAGF-19-0019_NDM-5 plasmid via electroporation. Growth and competition assays were performed as described above in the presence of 0.02% arabinose and 30 mg/L chloramphenicol to induce *bla*_OXA-181_ expression and to maintain the introduced plasmids, respectively. For the competition assay, the ratio of *E. coli* cells with and without *bla*_OXA-181_ was determined using PCR. Each culture was spread onto BHI agar plates containing 0.25 mg/L meropenem and 30 mg/L chloramphenicol. At least 20 colonies per condition were subjected to colony-direct PCR using a primer pair targeting the upstream and downstream regions of the pBAD18-cm cloning site (5′-GATTAGCGGATCCTACCTGAC-3′ and 5′-CTTCTCTCATCCGCCAAAAC-3′). Competition indices were calculated by dividing the number of pJBBDAGF-19-0019_NDM-5 transformants positive for a longer insert (i.e. *bla*_OXA-181_) by the total number of transformants.

### Nucleotide sequence accession numbers

The nucleotide sequence of strain JBEHAAB-19-0176 was deposited in DDBJ/ENA/GenBank under the BioSample accession number: SAMD00502403.

## Results and discussion

An isolate co-carrying *bla*_NDM-5_ and *bla*_OXA-181_, JBEHAAB-19-0176, was obtained from the faeces of an outpatient who had returned from a trip to Bangladesh and was diagnosed with *Campylobacter* enteritis. These carbapenemase genes are the most common and the third most common carbapenemase genes encountered in a hospital surveillance in Bangladesh, respectively;^22^ however, both genes have rarely been detected in Japan.^12^ Therefore, it is likely that the isolate was imported from Bangladesh. This strain belonged to ST648, a globally disseminated carbapenem-resistant clone and the first reported isolate with NDM-5 in the UK in 2011.^23^ Complete sequences showed that the isolates had a chromosome (5 224 427 bp) and five plasmids including the IncX3 plasmid pJBEHAAB-19-0176_NDM-OXA (63 152 bp) co-harbouring *bla*_NDM-5_, *bla*_OXA-181_ and a partial ColKP3 replicon. Details of the four other plasmids, namely pJBEHAAB-19-0176_1 (151 726 bp) harbouring IncFIB and IncFII replicons, pJBEHAAB-19-0176_2 (109 582 bp) harbouring IncFIB replicons, pJBEHAAB-19-0176_4 (59 260 bp) harbouring the IncI plasmid, and pJBEHAAB-19-0176_5 (3066 bp) harbouring the Col replicon, are listed in Table 1. The strain harboured resistance genes against aminoglycosides (*aadA2*), sulphonamides (*sul1*) and trimethoprim (*dfrA12*) on the chromosome. The quinolone resistance gene *qnrS1* was also located on the IncX3 plasmid, together with *bla*_NDM-5_ and *bla*_OXA-181_, whereas the macrolide resistance gene *mph*(A) was present on both the chromosome and IncFIB plasmids. Additionally, *bla*_CMY-141_ was located on an IncI (gamma) plasmid.

**Table 1.** Genomic information of *E. coli* JBEHAAB-19-0176

Replicon	Length (bp)	Inc type	Acquired antimicrobial resistance gene(s)	GenBank accession no.
Chromosome	5 224 427	ND	*mph*(A), *sul1*, *aadA2*, *dfrA12*, *qepA4*	AP028869
pJBEHAAB-19-0176_NDM-OXA	63 152	IncX3, ColKP3	*bla*_NDM-5_, *bla*_OXA-181_, *qnrS1*	AP028870
pJBEHAAB-19-0176_1	151 726	IncFIB, IncFII	*mph*(A), *erm*(B)	AP028871
pJBEHAAB-19-0176_2	109 582	IncFIB	ND	AP028872
pJBEHAAB-19-0176_4	59 260	IncI(gamma)	*bla*_CMY-141_	AP028873
pJBEHAAB-19-0176_5	3066	Col	ND	AP028874

ND, not detected.

**Figure 1.**
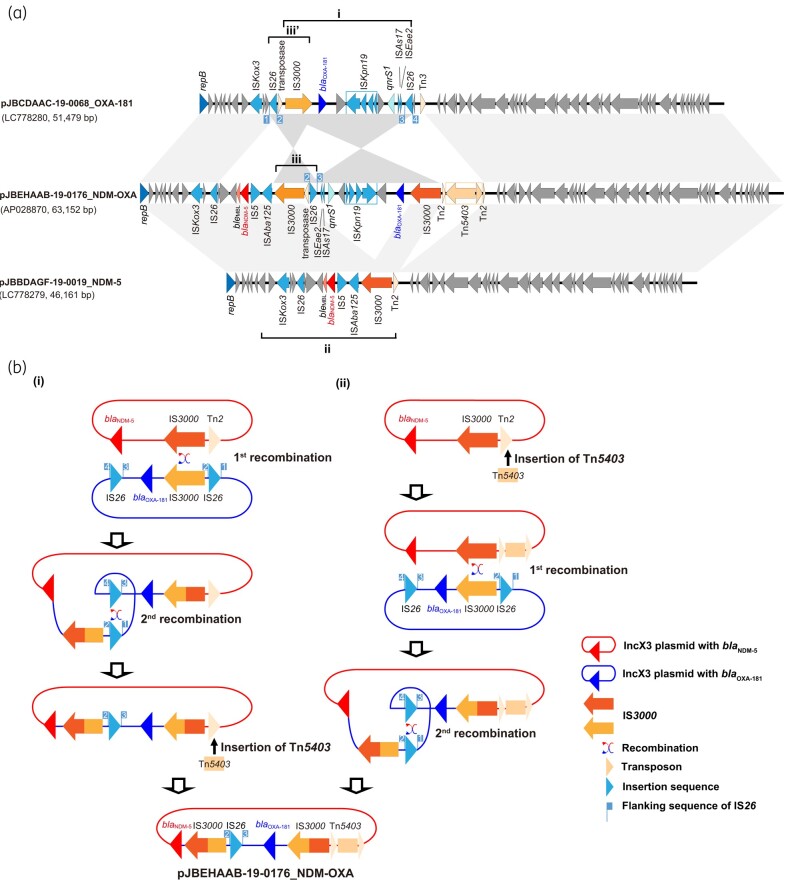
Genetic features of pJBEHAAB-19-0176_NDM-OXA and proposed models for its formation. (a) Linear comparison of the pJBEHAAB-19-0176_NDM-OXA plasmid with two other IncX3 plasmids harbouring *bla*_NDM-5_ or *bla*_OXA-181_. The coding sequences are represented by boxed arrows, and homologous regions (>99% identity) highlighted in grey shading. (b) Schematic diagram of pJBEHAAB-19-0176_NDM-OXA formation. The red line denotes an IncX3 plasmid with *bla*_NDM-5_, and the blue one denotes that with *bla*_OXA-181_. The red and orange arrows indicate IS*3000*, and the light blue arrows indicate IS*26*. Flags represent 8 bp flanking sequences of IS*26*. Wavy arrows show the recombination event.

IncX3 plasmids harbouring *bla*_NDM-5_ or *bla*_OXA-181_ have been frequently reported worldwide.^3–7^ We compared pJBEHAAB-19-0176_NDM-OXA with two representative IncX3 plasmids, a typical plasmid harbouring *bla*_NDM-5_ (pJBBDAGF-19-0019_NDM-5) and another harbouring *bla*_OXA-181_ (pJBCDAAC-19-0068_OXA-181) (Figure 1a), which were identified in the JBBDAGF-19-0019 and JBCDAAC-19-0068 *E. coli* isolates, respectively, in the JARBS-GNR surveillance.^12^ The three plasmids shared backbone genetic structures of the IncX3 plasmid, such as replication and conjugal transfer genes, which were 99.9% identical among the plasmids. A conjugation assay using a recipient strain *E. coli* ML4909 confirmed that pJBEHAAB-19-0176_NDM-OXA could be transferable with a transfer efficiency of 3.1 × 10^−2^, which was comparable to that of pJBBDAGF-19-0019_NDM-5 (1.4 × 10^−2^) and noticeably higher than that of pJBCDAAC-19-0068_OXA-181 (2.3 × 10^−4^). In addition to the IncX3 backbone, pJBEHAAB-19-0176_NDM-OXA had two genetic regions harbouring *bla*_OXA-181_ and *bla*_NDM-5_ (Figure 1a, i and ii), which were homologous to those found in pJBBDAGF-19-0019_NDM-5 and pJBCDAAC-19-0068_OXA-181, respectively. Among the three plasmids, two IS*3000* sequences and Tn*5403* were specific to pJBEHAAB-19-0176_NDM-OXA, whereas the other two plasmids had only one IS*3000* sequence and lacked Tn*5403*. The pJBEHAAB-19-0176_NDM-OXA and pJBBDAGF-19-0019_NDM-5 plasmids had similar gene synteny with identical *bla*_NDM-5_ regions; however, the *bla*_OXA-181_ region was located in opposite orientations between the two IS*3000*s in pJBCDAAC-19-0068_OXA-181. There was a cluster of three mobile genetic elements, namely IS*26*, a putative Tn*3*-family transposase, and IS*3000*, located on the left boundary of the inverted *bla*_OXA-181_ region (Figure 1a, iii). The same cluster was found in pJBCDAAC-19-0068_OXA-181 (Figure 1a, iii’). However, the IS*26* flanking sequences did not match between them, and the eight nucleotide sequences flanking the right side of IS*26* of cluster iii matched that of another IS*26* sequence on pJBCDAAC-19-0068_OXA-181 (flag 3 in Figure 1). These structural features suggest that pJBEHAAB-19-0176_NDM-OXA was formed through two recombination events (Figure 1b): (1) formation of a co-integrate between pJBBDAGF-19-0019_NDM-5 and pJBCDAAC-19-0068_OXA-181 via homologous recombination at IS*3000* sequences; followed by (2) a second recombination event at IS*26* sequences, resulting in excision of the IncX3 backbone of pJBCDAAC-19-0068_OXA-181. The Tn*5403* transposon insertion into Tn*2* could have occurred either after (Figure 1b, i) or before (Figure 1b, ii) these events. Both IS*3000* and IS*26* are known for their crucial roles in forming self-transmissible mobile genomic elements through a replicative mechanism and co-integrate formation during transposition.^24–26^ Our hypothesis assumes the coexistence of the two plasmids; however, it is unclear how they can coexist in a bacterial cell as they cannot be stably maintained due to plasmid incompatibility. One possible explanation is that an IncX3 plasmid carrying one gene was introduced into the bacteria along with the IncX3 plasmid carrying another gene via conjugal transfer, resulting in transient coexistence of the two plasmids. Considering the remarkably higher transfer efficiency of the plasmid with *bla*_NDM-5_ than that with *bla*_OXA-181_ found in this study (almost 100-fold), it is likely that the *bla*_NDM-5_-carrying plasmid was introduced into an organism already carrying *bla*_OXA-181_.

Dual carriage of carbapenemase genes can contribute to high carbapenem resistance compared with that of organisms with single carriage.^27^ We generated the *E. coli* HST08 transformants carrying one of three IncX3 plasmids to investigate the phenotypic differences and potential benefits of co-carrying *bla*_NDM-5_ and *bla*_OXA-181_ compared with when either gene is carried alone. Antimicrobial susceptibility testing via a broth microdilution method showed that for the pJBEHAAB-19-0176_NDM-OXA transformants, the carbapenem MICs were identical to those of the pJBBDAGF-19-0019_NDM-5 transformants (Table 2). Interestingly, the MICs of ampicillin, piperacillin and cefpodoxime were 2-fold higher in the pJBEHAAB-19-0176_NDM-OXA transformants than in the pJBBDAGF-19-0019_NDM-5 transformants, indicating that the additional carriage of *bla*_OXA-181_ could increase the MICs of these antibiotics. Although pJBCDAAC-19-0068_OXA-181 showed 16 times lower carbapenem MICs than those of the other two transformants, it showed a >16 times higher MIC than that of the host (*E. coli* HST08).

**Table 2.** Antimicrobial susceptibility patterns of *bla*_NDM-5_- and/or *bla*_OXA-181_-harbouring isolates and transformants

Antimicrobial agents	MICs (mg/L)						
	JBCDAAC-19-0068_OXA-181	JBEHAAB-19-0176_NDM-OXA	JBBDAGF-19-0019_NDM-5	HST:: pJBCDAAC-19-0068_OXA-181	HST:: pJBEHAAB-19-0176_NDM-OXA	HST:: pJBBDAGF-19-0019_NDM-5	HST08
Meropenem^a^	0.5	128	64	2	32	32	≤0.125
Imipenem^a^	2	64	64	2	32	32	≤0.125
Ampicillin^a^	2048	32 768	16 384	1024	8192	4096	≤4
Piperacillin^a^	2048	>4096	>4096	128	1024	512	1
Cefpodoxime^a^	2048	4096	2048	1	256	128	0.5
Doripenem	≤0.5	>8	>8	1	>8	>8	≤0.5
Ampicillin/sulbactam	32/16	>32/16	>32/16	32/16	>32/16	>32/16	≤4/2
Piperacillin/tazobactam	64	>64	>64	8	64	>64	≤4
Cefazolin	>16	>16	>16	16	>16	>16	≤1
Cefoperazone/sulbactam	32/16	>32/16	>32/16	≤8/4	>32/16	>32/16	≤8/4
Cefoxitin	≤8	>32	>32	8	>32	>32	≤8
Cefmetazole	2	>32	>32	4	16	16	≤0.5
Cefotetan	≤1	>32	>32	4	>32	>32	≤1
Flomoxef	≤8	>32	>32	≤8	>32	>32	≤8
Ceftazidime	16	>128	>128	≤0.5	>128	>128	≤0.5
Ceftazidime/clavulanic acid	2/4	>32/4	>32/4	0.12/4	>32/4	>32/4	0.12/4
Cefotaxime	>128	>128	>128	≤0.5	>128	>128	≤0.5
Cefotaxime/clavulanic acid	>32/4	>32/4	>32/4	0.25/4	>32/4	>32/4	≤0.125/4
Ceftriaxone	>64	>64	>64	≤0.5	>64	>64	≤0.5
Cefepime	>32	>32	>32	≤1	32	>32	≤1
Cefozopran	>16	>16	>16	2	>16	>16	≤1
Aztreonam	64	64	2	≤0.5	≤0.5	≤0.5	≤0.5
Gentamicin	≤1	2	>8	≤1	≤1	≤1	≤1
Tobramycin	2	2	>8	≤1	≤1	≤1	≤1
Amikacin	≤4	8	>32	≤4	≤4	≤4	≤4
Levofloxacin	≤0.5	>8	>8	1	1	≤0.5	≤0.5
Ciprofloxacin	0.5	>4	>4	1	0.5	≤0.25	≤0.25
Minocycline	2	≤1	8	≤1	≤1	≤1	≤1
Trimethoprim/sulfamethoxazole	>2/38	>2/38	>2/38	≤1/19	≤1/19	≤1/19	≤1/19
Fosfomycin	16	16	>16	≤4	≤4	≤4	≤4
Chloramphenicol	≤8	16	>16	≤8	≤8	≤8	≤8
Colistin	≤1	≤1	≤1	≤1	≤1	≤1	≤1
Tigecycline	≤0.25	≤0.25	≤0.25	≤0.25	≤0.25	≤0.25	≤0.25

^a^MIC was determined using the broth microdilution method.

**Figure 2.**
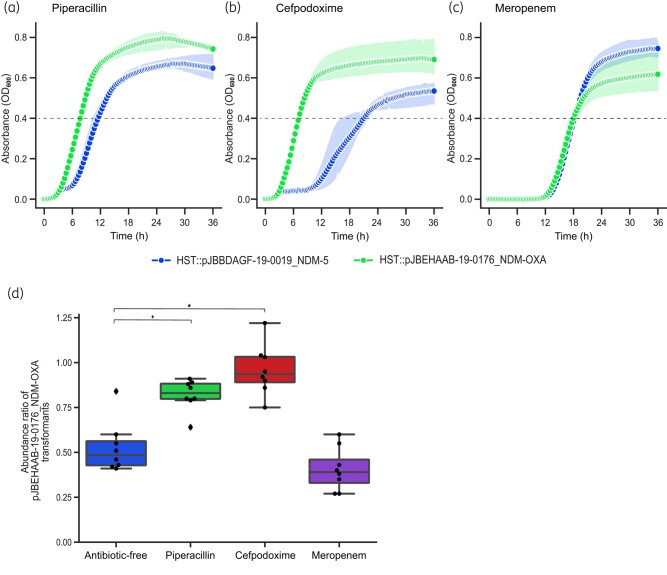
The transformant carrying a plasmid co-harbouring *bla*_NDM-5_ and *bla*_OXA-181_ outcompetes one carrying a plasmid with only *bla*_NDM-5_ in the presence of piperacillin and cefpodoxime. Bacterial growth curves of pJBEHAAB-19-0176_NDM-OXA (HST::pJBEHAAB-19-0176_NDM-OXA, green) and pJBBDAGF-19-0019_NDM (HST::pJBBDAGF-19-0019_NDM-5, blue) transformants. OD_600_ was monitored in the presence of 64 mg/L piperacillin (a), 64 mg/L cefpodoxime (b) or 128 mg/L meropenem (c). The experiment was repeated three times. The pJBEHAAB-19-0176_NDM-OXA and pJBBDAGF-19-0019_NDM-5 transformants were mixed and cultured in the presence or absence of 128 mg/L piperacillin, 64 mg/L cefpodoxime and 16 mg/L meropenem. The bacterial counts were measured 24 h after inoculation, and the abundance ratio of pJBEHAAB-19-0176_NDM-OXA transformants calculated (d). The experiment was repeated eight times. Error bars represent standard deviations. Asterisks show significant differences (Steel test, *P* < 0.05).

We further investigated the growth rates, i.e. the time to reach the exponential phase, of the two transformants through measuring changes in the OD_600_ over time in the presence of different antibiotics. Notably, the growth rate of the pJBEHAAB-19-0176_NDM-OXA transformant was higher than that of the pJBBDAGF-19-0019_NDM-5 transformant in the presence of piperacillin and cefpodoxime (Figure 2a and b). In contrast, both transformants showed comparable growth rates in the presence of meropenem and other β-lactams tested (Figure 2c and Figure S1, available as Supplementary data at *JAC-AMR* Online). The pJBEHAAB-19-0176_NDM-OXA transformants reached OD_600_ = 0.4 at 7.6 and 7.0 h after being inoculated in the presence of piperacillin and cefpodoxime, respectively. In contrast, the pJBBDAGF-19-0019_NDM-5 transformant took longer to reach OD_600_ = 0.4 (11.6 and 21.0 h, respectively) under the same conditions. These results suggest that the *bla*_NDM-5_/*bla*_OXA-181_ co-carrier had a growth advantage in the presence of these antibiotics, particularly cefpodoxime, compared with that of the *bla*_NDM-5_ single carrier. We tested this using a competition assay in which pJBEHAAB-19-0176_NDM-OXA and pJBBDAGF-19-0019_NDM-5 transformants were mixed and incubated in the presence of the above antibiotics. The bacteria were counted 24 h after inoculation (Figure 2d), and results showed that the growth of these transformants was almost comparable in the absence of any drugs and in the presence of meropenem. In contrast, the pJBEHAAB-19-0176_NDM-OXA transformant outcompeted the pJBBDAGF-19-0019_NDM-5 transformant in the presence of piperacillin and cefpodoxime, suggesting that the additional carriage of *bla*_OXA-181_ confers a competitive advantage over *bla*_NDM-5_ single carriers under these conditions.

**Figure 3.**
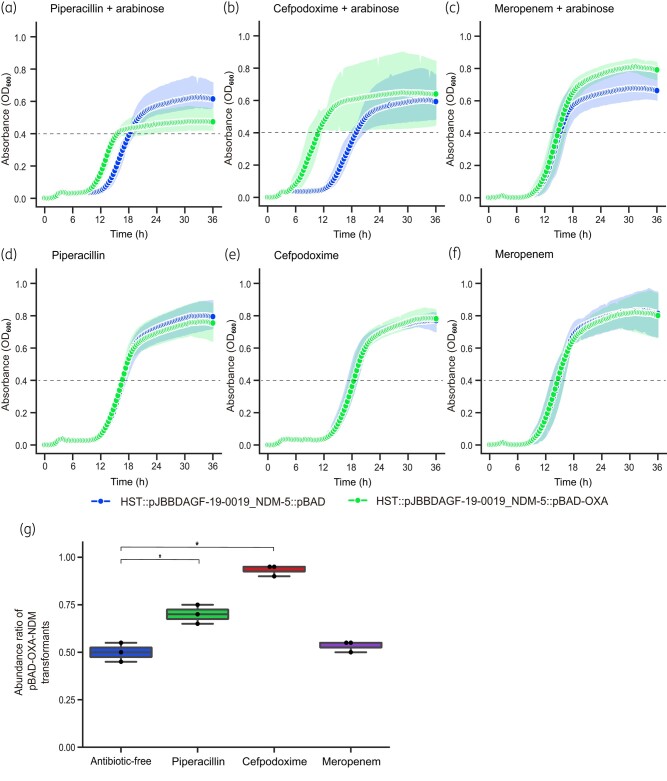
Expression of *bla*_OXA-181_ confers a growth advantage to the transformant carrying *bla*_NDM-5_ in the presence of piperacillin and cefpodoxime. The OD_600_ of the pJBBDAGF-19-0019_NDM-5 transformant carrying the pBAD18-cm empty vector (HST::pJBBDAGF-19-0019_NDM-5::pBAD, blue) or the one harbouring *bla*_OXA-181_ (HST::pJBBDAGF-19-0019_NDM-5::pBAD-OXA, green) were monitored with (a–c) or without (d–f) 0.02% arabinose in the presence of 64 mg/L piperacillin (a, d), 64 mg/L cefpodoxime (b, e) or 128 mg/L meropenem (c, f). (g) The pJBBDAGF-19-0019_NDM-5 transformant carrying pBAD18-cm or the one harbouring *bla*_OXA-181_ (pBAD-OXA) were mixed and cultured in the presence or absence of 128 mg/L piperacillin, 64 mg/L cefpodoxime and 16 mg/L meropenem—0.02% arabinose was included in all test conditions. The abundance ratio of the transformants carrying pBAD-OXA was determined 24 h after inoculation. The experiment was repeated three times. Error bars represent standard deviations. Asterisks show significant differences (Tukey’s test, *P* < 0.05).

To test this notion further, we compared the growth rate of pJBBDAGF-19-0019_NDM-5 transformants carrying an arabinose-inducible expression vector with (pBAD-OXA-181) or without (pBAD) *bla*_OXA-181_. When the experiment was performed with piperacillin and cefpodoxime, the growth rate of the transformant with pBAD-OXA-181 was faster than that with pBAD in the presence of arabinose (Figure 3a and b). Similar differences were not observed in the absence of arabinose (Figure 3d and e) and in the condition when meropenem was used instead of piperacillin/cefpodoxime (Figure 3c and f). In addition, the pJBBDAGF-19-0019_NDM-5 transformant carrying pBAD-OXA-181 outcompeted the one carrying pBAD in the presence of arabinose and piperacillin or cefpodoxime, but not meropenem (Figure 3g). Thus, the additional expression of *bla*_OXA-181_ was sufficient to confer a growth advantage in the presence of piperacillin and cefpodoxime. OXA-48 efficiently hydrolyses piperacillin, and OXA-181 shows similar substrate specificity.^28,29^ Therefore, it is probable that OXA-181 can hydrolyse piperacillin, and the additional presence of its encoding gene provides additional piperacillin hydrolytic activity to the bacteria. In contrast, it is unclear why additional carriage of *bla*_OXA-181_ provides a growth advantage in the presence of cefpodoxime because a subset of OXA-48 enzymes, including OXA-181, is incapable of hydrolysing extended-spectrum cephalosporins.^6^ This issue should be addressed in future studies.

From the perspective of the molecular epidemiology of CPE, isolates co-carrying the two carbapenemase genes, *bla*_NDM_ and *bla*_OXA-48_-like, have been found in various genotypes of Enterobacterales and have been increasingly reported worldwide.^10,30–33^ It was also observed that isolates co-carrying the two carbapenemase genes, *bla*_NDM-1_ and *bla*_OXA-232_ or *bla*_OXA-181_, emerged in three different STs of *Klebsiella pneumoniae* during a 4 year hospital surveillance, with a concomitant decrease in *bla*_NDM-1_-carrying isolates.^34^ The present study provides a clue to understanding the changing epidemiology of CPE. Piperacillin and cefpodoxime could be selective agents for the emergence of isolates co-carrying *bla*_NDM_ and *bla*_OXA-48_-like genes. Meanwhile, it is conceivable that there are other advantages conferred by double carriage of these genes, which were not explored in this study. In addition, it should be noted that the changing epidemiology can result from many other factors, including characteristics of the host bacteria and plasmids carrying these genes, carriage of other antimicrobial resistance genes, and antimicrobial use.

In conclusion, we identified an IncX3 plasmid co-carrying dual carbapenemase genes in a national surveillance study using short- and long-read WGS. The plasmid was formed via the convergence of two highly disseminated plasmids in an endemic region and then imported to Japan. Due to the global spread of such problematic antimicrobial-resistant plasmids, continued surveillance and efforts to limit their further spread are warranted.

## Supplementary Material

dlae073_Supplementary_Data

## Supplementary data

Figure S1 is available as Supplementary data at *JAC-AMR* Online.
