# Supplementary material for: Emergence of an IncX3 plasmid co-harbouring the carbapenemase genes blaNDM-5 and blaOXA-181
Source: JAC Antimicrob Resist. 2024 May 13;6(3):dlae073. doi: 10.1093/jacamr/dlae073 (PMC11089413; doi:10.1093/jacamr/dlae073)
Supplement: dlae073_Supplementary_Data [file dlae073_supplementary_data.docx]

**Figure S1**

Bacterial growth curves of pJBEHAAB-19-0176_NDM-OXA (HST::pJBEHAAB-19-0176_NDM-OXA, green) and pJBBDAGF-19-0019_NDM-5 (HST::pJBBDAGF-19-0019_NDM-5, blue) transformants. Optical density at 600 (OD_600_) was monitored in the presence of 2048 mg/L Ampicillin (a), 512 mg/L cefazolin (b), 512 mg/L cefoxitin (c), 256 mg/L flomoxef (d), 512 mg/L ceftazidime (e), 512 mg/L cefotaxime (f), 128 mg/L cefepime (g), or 128 mg/L imipenem (h). The experiment was repeated three times. Error bars show standard deviation.
